# Pressure-induced magnetic transitions with change of the orbital configuration in dimerised systems

**DOI:** 10.1038/srep25831

**Published:** 2016-05-18

**Authors:** Dmitry M. Korotin, Vladimir I. Anisimov, Sergey V. Streltsov

**Affiliations:** 1 Institute of Metal Physics, S. Kovalevskoy St. 18, 620990 Yekaterinburg, Russia; 2 Department of theoretical physics and applied mathematics, Ural Federal University, Mira St. 19, 620002 Yekaterinburg, Russia

## Abstract

We suggest a possible scenario for magnetic transition under pressure in dimerised systems where electrons are localised on molecular orbitals. The mechanism of transition is not related with competition between kinetic energy and on-site Coulomb repulsion as in Mott-Hubbard systems, or between crystal-field splitting and intra-atomic exchange as in classical atomic spin-state transitions. Instead, it is driven by the change of bonding-antibonding splitting on part of the molecular orbitals. In the magnetic systems with few half-filled molecular orbitals external pressure may result in increase of the bonding-antibonding splitting and localise all electrons on low-lying molecular orbitals suppressing net magnetic moment of the system. We give examples of the systems, where this or inverse transition may occur and by means of *ab initio* band structure calculations predict that it can be observed in *α*−MoCl_4_ at pressure *P* ~ 11 GPa.

In spite of a long history of the magnetic phenomena investigation, magnetism remains one of the most attractive subjects for a research due to both extensive technological applications of different magnetic materials and its exceptional fundamental importance for the science, which led to development of such conceptions and ideas as electromagnetism, spin etc. Special attention is paid to the study of formation or destruction of the local magnetic moments. There are several mechanisms responsible for this.

In strongly correlated materials local magnetic moments, which usually exist in the insulating phase, are destroyed with Mott-Hubbard transition to metallic state driven by competition of the kinetic energy (given by hopping parameter *t*) and Coulomb repulsion *U*^1^,^2^. This type of transitions can be found mainly in transition metal (TM) compounds^3^, while for 4*f*–5*f* systems the Kondo effect^4^ may lead to decrease of the measured local magnetic moment, due to screening by conduction electrons, as it occurs in metallic Ce^5^,^6^. The screening is defined by the hybridization between localised *f* and band *s*, *p*, *d* states, which can be controlled by, e.g., pressure. Another mechanism of the magnetic moment suppression is the spin-state transition, when an increase of the crystal field splitting between *t*_2*g*_ and *e*_*g*_ bands due to external pressure or decrease of the temperature (resulting in the lattice contraction) leads to violation of the first Hund’s rule maximizing the spin moment of an ion. This phenomenon is quite important in geophysics, since many materials constituting Earth’s crust and mantle, as e.g. (MgFe)O^8^, MnO^9^, or Na(Fe, Cr)Si_2_O_6_^10^ do show such transitions.

All these effects are, however, related to the suppression of the magnetic moments on some particular ions, while there are situations, when these moments are formed not on atomic, but on molecular orbitals, as it occurs in (Na, K)O_2_^11^, SrRu_2_O_6_^12^, Ba_4_Ru_3_O_10_^7^, Nb_2_O_2_F_3_^13^ and in many other compounds. In the present paper we show that an external pressure may also induce magnetic transition in the systems with atomic complexes such as dimers, trimers etc., where electrons occupy molecular orbitals. This transition from magnetic to nonmagnetic state is possible, when there are few nearly degenerate half-filled molecular orbitals. External pressure may increase splitting and stabilise all electrons on low-lying molecular orbitals. Detailed study of this effect was performed for one of such dimerised system: *α*−MoCl_4_. By means of *ab initio* band structure calculations we estimated the critical pressure for the transition to nonmagnetic state in this compound.

## General Treatment

**Figure 1:**
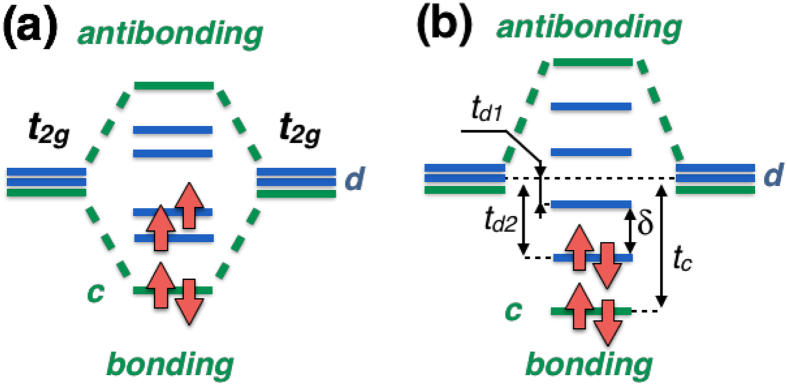
Two possible electronic configurations with finite and zero spin moment in case of a dimer with three (*t*_2*g*_) orbitals and two electrons per site. It is supposed that there are two different sets of orbitals in the system: *c* orbitals (green) with larger bonding-antibonding splitting, given by hopping parameter *t*_*c*_, and *d* orbitals (blue). If hopping for one of the *d* orbitals is much larger than for another *t*_*d*2_ ≫ *t*_*d*1_, when nonmagnetic state is realized in the system.

We start with an isolated dimer. Transition metals are in the ligand octahedra and these octahedra share their edges or faces (metal-metal distances in the “common corner” geometry are usually too large for the formation of molecular orbitals). There are two types of orbitals in such geometries, which we will denote as *c* and *d*. The *c* orbitals have a direct overlap, characterized by hopping parameter *t*_*c*_, with neighbouring transition metal (the *a*_1*g*_ orbitals in the “common face”^14^,^15^ and the *xy* orbitals in the “common edge” geometry^16^), which results in a large bonding-antibonding splitting, 2*t*_*c*_. If there are more than one electron per site and *t*_*c*_ is large enough (with respect to Hund’s rule coupling *J*_*H*_), the bonding molecular orbital is fully occupied and corresponding electrons do not contribute to the total magnetic moment of the dimer^17^. So that the magnetization is defined by other, *d*, electrons, localised on the 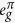 (for face-sharing) or *xz*/*yz* (for edge-sharing) orbitals, as shown in Fig. 1(a).

These two *d* orbitals (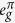 for face-sharing and *xz*/*yz* for edge-sharing geometry) are not directed to each other, so that corresponding hoppings (*t*_*d*1_, *t*_*d*2_) ≪ *t*_*c*_. It is important for us here that *t*_*d*1_ and *t*_*d*2_ can also be very different, since typically the TMO_6_ octahedra are strongly distorted in the dimerised systems. Applying external pressure one increases all the bonding-antibonding splittings, given by hopping parameters *t*_*d*1_, *t*_*d*2_, and *t*_*c*_, and may suppress net magnetization, even if there was nonzero magnetic moment at ambient conditions. In a some sense this effect reminds classical atomic spin-state transition^1^, but instead of the crystal-field splitting here bonding-antibonding splitting between molecular orbitals competes with the Hund’s rule coupling.

It is easier to illustrate this general picture on a particular example. Let us consider dimerised system with two electrons per site, *t*_*d*1_ ≠ *t*_*d*2_, and (*t*_*d*1_, *t*_*d*2_) ≪ *t*_*c*_. Then two out of four electrons will occupy *c* bonding orbitals, while the rest two electrons provide magnetic moment, see Fig. 1(a). If these *d*1 and *d*2 orbitals are molecular orbitals then there is a gain in intra-atomic exchange energy for spin triplet (ferromagnetic) state with respect to spin singlet (antiferromagnetic) one. In the ionic limit taking into account intra-atomic Hund’s rule as 
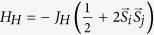
 and neglecting on-site Coulomb repulsion *U* (and hence modification of the ground state wave function from molecular orbital-like to Heitler-London) one may find that the energy of this state will be


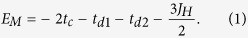


Applying external pressure we increase all the hopping parameters *t*_*c*_, *t*_*d*1_, and *t*_*d*2_, so that finally one may end up with the situation, when not only *c*, but also one of the *d* molecular orbitals is completely filled, as shown in Fig. 1(b). The energy of this nonmagnetic state will be


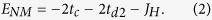


Comparing last two equations one finds that the transition to the nonmagnetic state is expected, when


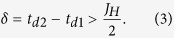


In real materials the situation, however, can be much more complicated. Mentioned above effect of the Hubbard *U* does not simply renormalize *J*_*H*_, but changes energetics of the bonding orbitals, which is defined solely by corresponding hopping parameter *t* in the absence of *U* and by *t*^2^/*U* in the large *U* limit. In addition the on-site energies of the *d* orbitals can be very different due to strong distortions of the TMO_6_ octahedra. However, qualitative picture is rather general: having magnetic dimerised system with few degenerate or nearly degenerate half-filled *d* molecular orbitals one may expect to have a transition to nonmagnetic state under external or due to internal (chemical) pressure. In order to check this effect we performed *ab initio* band structure calculations for *α*−MoCl_4_, which fulfills aforementioned conditions.

## Pressure-induced Magnetic Transition in *α*−MoCl_4_

**Figure 2:**
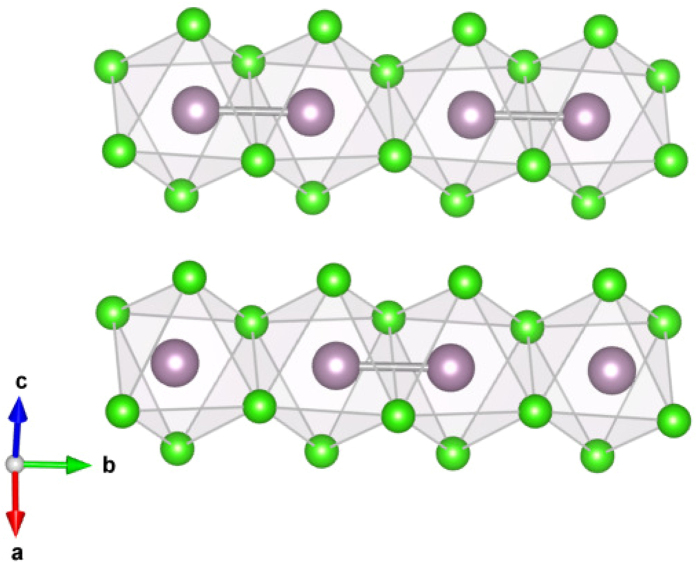
Crystal structure of *α*−MoCl_4_. Green balls denote Cl, violet - Mo ions. Mo-Mo dimers are shown by thick violet lines.

The *α*−MoCl_4_ crystalizes in the NbCl_4_ structure consisting of Mo-Mo dimers^18^, see Fig. 2. Mo^4+^ has 4*d*^2^ electronic configuration and at ambient conditions this material is paramagnetic with positive Curie-Weiss temperature ~220 K^17^, which presumes net ferromagnetic exchange coupling. The effective magnetic moment is ~0.85 − 0.93*μ*_*B*_^18^,^19^, much smaller than *μ*_*eff*_ = 2.82 *μ*_*B*_ expected for isolated Mo^4+^ ion having *S* = 1.

**Figure 3:**
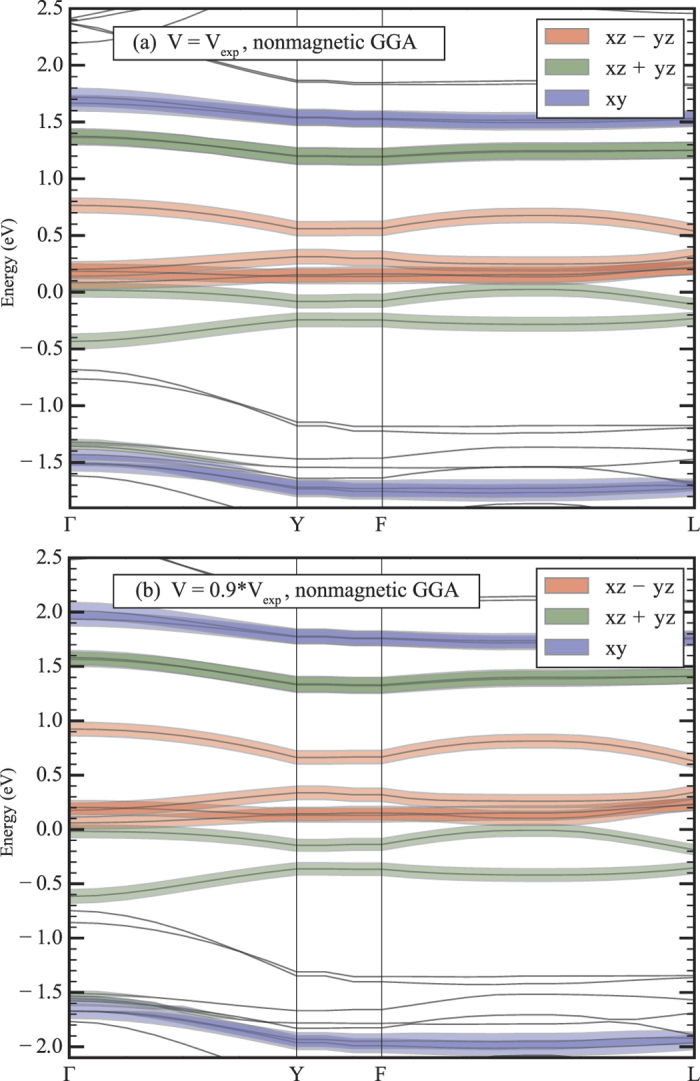
Band structure of *α*−MoCl_4_ as obtained in the nonmagnetic GGA calculations for experimental volume and for the volume below magnetic transition. Contribution of Mo 4*d* orbitals is shown by different colours. Since there are two Mo-Mo dimers in the unit cell each band is double degenerate. Fermi energy is in zero.

**Figure 4:**
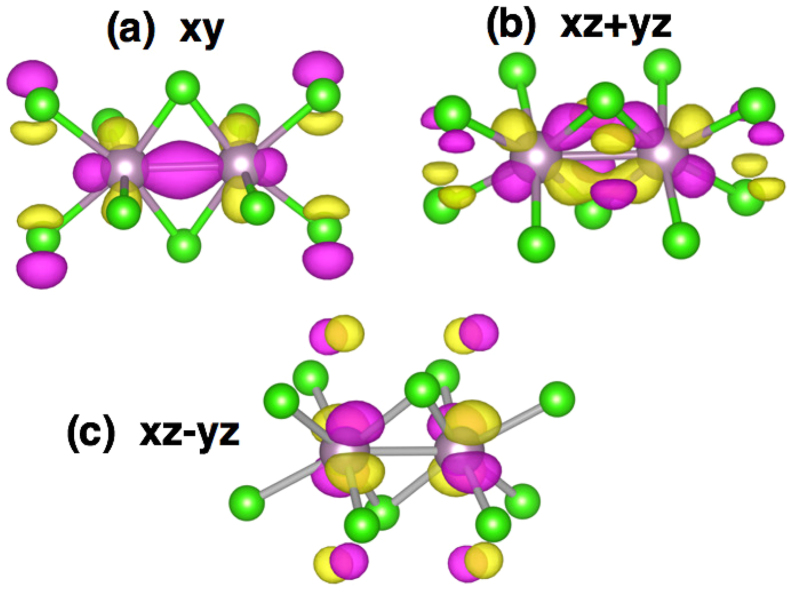
Charge density plot obtained in the nonmagnetic GGA + U calculations for experimental volume. Colours for atoms are the same as in Fig. 2, pink and yellow indicate different signs of the charge density.

Suppression of the magnetic moment is related to orbital-selective effect in dimerised systems^17^,^18^. Each Mo is in the Cl_6_ octahedron and two neighbouring octahedra share their edges forming a dimer. As a result there has to be a strong bonding-antibonding splitting for the *xy* orbitals, which play a role of the *c* orbitals (here and below all notations are with respect to the local coordinate system, where axis are directed to Cl, and *x* and *y* are in the plane of common edge and short Mo-Mo bond). Bonding *xy* orbitals are fully occupied and this explains experimentally observed partial suppression of the magnetic moment in this system at ambient conditions. This strong splitting ~3.2 eV is clearly seen from the nonmagnetic band structure (Fig. 3(a)), obtained in the generalized gradient approximation (GGA). In Fig. 4(a) we plotted the charge density corresponding to these bonding *xy* orbitals.

The *xz* and *yz* orbitals also form molecular orbitals. These are *d*1 and *d*2 orbitals in the notations of the previous section. This is clear that effective *d* − *d* hopping via Cl *p*_*z*_ orbital is the same for *xz* and *yz* orbitals centered on different sites, but one may also maximize direct *d* − *d* hopping constructing the *xz* + *yz* orbital, see Fig. 4(b), so that systems gains maximum kinetic energy localising electrons on these *xz* + *yz* and *xz* − *yz* orbitals. Very similar situation is observed in Li_2_RuO_3_^21^. The bonding-antibonding splitting for the *xz* + *yz* molecular orbitals is ~1.6 eV, while for *xz* − *yz* it is much smaller, ~0.2 eV.

In order to take into account strong Coulomb correlations we performed the GGA + U calculations. Constrained RPA (cRPA) calculations for metallic Mo give *U* − *J*_*H*_ ~ 3 eV^22^. One may think that metallic Mo is very different from MoCl_4_ and Hubbard *U* in a chloride can be much larger resulting to Mott-Hubbard physics. However, an estimation of *U* using constrained GGA method within the Wannier function formalism^23^ for *α* − MoCl_4_ gives *U*~2.9 eV, so that one may use the cRPA result for the GGA + U calculation. The local magnetic moment in the GGA + U was found to be *m*_*tot*_ = 0.85 *μ*_*B*_/Mo. Analysis of the occupation matrix shows that this moment is mainly due to the *xz* + *yz* and *xz* − *yz* orbitals: *m*_*xz*+*yz*_ = 0.29*μ*_*B*_ and *m*_*xz*−*yz*_ = 0.36*μ*_*B*_. Because of a large spatial extension of the Mo 4*d* orbitals substantial portion of the spin density is on the ligands, *m*_*Cl*_ = 0.07*μ*_*B*_/Cl.

**Figure 5:**
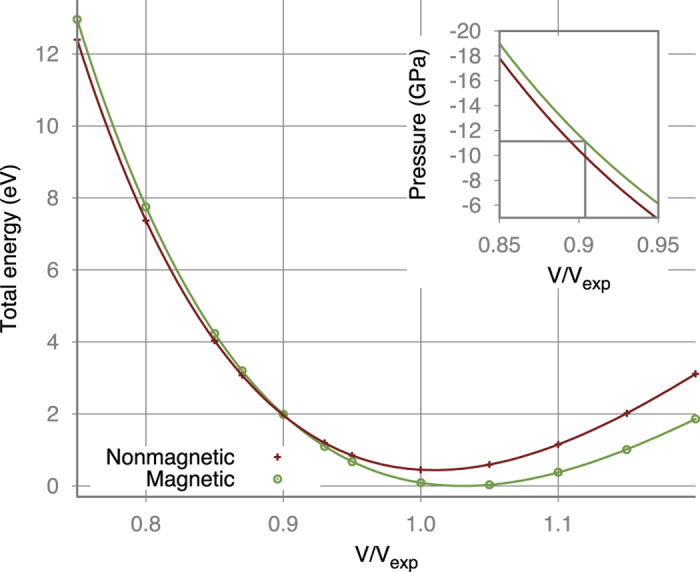
Total energy dependence on volume for ferromagnetic and nonmagnetic configurations as obtained in the GGA + U calculations for *α*−MoCl_4_. One may see in the inset that magnetic transition occurs at critical pressure *P*_*c*_~11 GPa.

Increasing pressure we induce magnetic transition, as it was described above in details. We studied this transition by the total energy (*E*) GGA + U calculations for ferromagnetic and nonmagnetic configurations for several volumes (*V*). Corresponding *E*(*V*) dependencies are shown in Fig. 5. The first order transition with collapse of the volume was found at critical pressure *P*_*c*_ = 11.2 GPa, which was estimated by fitting *E*(*V*) with the fifth order polynomial and finding its derivative^10^,^24^,^25^. Analysis of the occupation matrix shows that orbitals configuration indeed changes in the nonmagnetic phase, where four electrons occupy *xy* and *xz* + *yz* bonding orbitals, as it is shown in Fig. 1(b).

Thus, we see that the magnetic transition proposed in the previous section basing on quite general arguments does occurs in the GGA + U calculation for the real material, *α*−MoCl_4_. One may also argue that such transition can be realized in many other different systems, e.g. in WCl_4_^26^ or Nb_2_O_2_F_3_^13^. Moreover, an inverse transition from nonmagnetic to ferromagnetic state under tensile stress is also possible. It would be interesting to study whether such transition can be observed, e.g. in MoO_2_ or WO_2_ films grown on the substrates with larger inter-atomic distances.

It is also exciting that very similar transition seems to occur in famous half-metallic CrO_2_. At ambient conditions this compound is ferromagnetic and has the rutile crystal structure, where neighbouring CrO_6_ octahedra share their edges^27^. Main mechanism of the ferromagnetism is double exchange, when itinerant *xy* electrons make localised *xz*/*yz* electrons to have the same spin projection^28^. On a language of an isolated dimer this would correspond to the situation when *xy* is *c* and *xz*/*yz* are *d* orbitals and having two electrons per Cr site we fully occupy *xy* (*c*) orbital and leave *xz*/*yz* (*d*) orbitals half-filled to fulfill Hund’s rule. The LDA + U calculations show that this is exactly what is going on in CrO_2_^28^. However, detailed band structure calculations shows that CrO_2_ undergoes structural phase transition to dimerised phase at *P*~70 GPa and turns out to be nonmagnetic^29^. This strongly reminds pressure-induced transition in *α*−MoCl_4_ discussed in the present paper.

## Conclusions

To sum up in the present paper we considered the dimerised transition metal compounds with degenerate (or nearly degenerate) half-filled magnetic molecular orbitals and showed that the pressure-induced magnetic transition is possible in this case. This transition to nonmagnetic state is related to the change of the orbital configuration and results in a strong suppression of the magnetic moment of the system. Using band structure calculations we checked that this transition does occurs in one of such systems: *α*−MoCl_4_ and argue that it can be related to stabilization of nonmagnetic state in CrO_2_ under high pressure.

## Methods

All calculations in this work were performed with Quantum-ESPRESSO package^30^ that implements the ultrasoft pseudopotential formalism in plane-waves basis. The exchange-correlation potential was taken in the form proposed in ref. 31. A kinetic energy cutoff for the plane-wave expansion of the electronic states was set to 45 Ry. Reciprocal space integration were done on a regular 8 × 8 × 8 *k-*points grid in the irreducible part of the Brillouin zone. In order to check reliability of pseudopotential method we calculated *δE* = *E*_*FM*_ − *E*_*NM*_ at *V* = 0.8 *V*_exp_ using our ultrasoft pseudopotentials and the projector augmented-wave (PAW) method^32^. The difference in *δE* in these two calculations was found to be less than 1%. The crystal structure was taken from ref. 18.

## Additional Information

**How to cite this article**: Korotin, D. M. *et al.* Pressure-induced magnetic transitions with change of the orbital configuration in dimerised systems. *Sci. Rep.* **6**, 25831; doi: 10.1038/srep25831 (2016).

## References

[b1] Khomskii D. I. Transition Metal Compounds (Cambridge University Press, 2014).

[b2] Georges A., Krauth W. & Rozenberg M. J. Dynamical mean-field theory of strongly correlated fermion systems and the limit of infinite dimensions. Reviews of Modern Physics 68, 13–125 (1996).

[b3] Imada M., Fujimori A. & Tokura Y. Metal-insulator transitions. Reviews of Modern Physics 70, 1039–1263 (1998).

[b4] Hewson A. C. The Kondo Problem to Heavy Fermions. Cambridge Studies in Magnetism (Cambridge University Press, 1997).

[b5] Koskenmaki D. & Gschneidner K. No Title. In Handbook on the Physics and Chemistry of Rare Earths, chap. 4 (Elsevier, Amsterdam, 1978).

[b6] Streltsov S. V. *et al.* Magnetic susceptibility of cerium: An LDA+DMFT study. Physical Review B 85, 195109 (2012).

[b7] Streltsov S. V. & Khomskii D. I. Unconventional magnetism as a consequence of the charge disproportionation and the molecular orbital formation in Ba4Ru3O10. Phys. Rev. B 86, 064429 (2012).

[b8] Skorikov N. A., Shorikov A. O., Skornyakov S. L., Korotin M. A. & Anisimov V. I. Mechanism of magnetic moment collapse under pressure in ferropericlase. Journal of Physics: Condensed Matter 27, 275501 (2015). 26086296 10.1088/0953-8984/27/27/275501

[b9] Kuneš J., Lukoyanov A. V., Anisimov V. I., Scalettar R. T. & Pickett W. E. Collapse of magnetic moment drives the Mott transition in MnO. Nat Mater 7, 198–202 (2008). 18246073 10.1038/nmat2115

[b10] Streltsov S. V. & Skorikov N. A. Spin-state transitions in CaFeSi$_2$O$_6$ and NaFeSi$_2$O$_6$ under pressure. Phys. Rev. B 83, 214407 (2011).

[b11] Solovyev I. V., Pchelkina Z. V. & Mazurenko V. V. Magnetism of sodium superoxide. CrysEngComm 16, 522 (2014).

[b12] Streltsov S., Mazin I. I. & Foyevtsova K. Localized itinerant electrons and unique magnetic properties of SrRu2O6. Phys. Rev. B 92, 134408 (2015).

[b13] Tran T. T. *et al.* Nb2O2F3: A Reduced Niobium (III/IV) Oxyfluoride with a Complex Structural, Magnetic, and Electronic Phase Transition. Journal of the American Chemical Society 137, 636–639 (2015). 25581015 10.1021/ja511745q

[b14] Kugel K. I., Khomskii D. I., Sboychakov A. O. & Streltsov S. V. Spin-orbital interaction for face-sharing octahedra: Realization of a highly symmetric SU(4) model. Physical Review B 91, 155125 (2015).

[b15] Khomskii D. I., Kugel K. I., Sboychakov A. O. & Streltsov S. V. Role of Local Geometry in the Spin and Orbital Structure of Transition Metal Compounds. Journal of Experimental and Theoretical Physics 122, 484 (2016).

[b16] Goodenough J. B. Magnetism and the Chemical Bond (Interscience publishers, New York-London, 1963).

[b17] Streltsov S. V. & Khomskii D. I. Orbital-dependent singlet dimers and orbital-selective Peierls transitions in transition-metal compounds. Phys. Rev. B 89, 161112 (2014).

[b18] Kepert D. & Mandyczewsky R. a-Molybdenum Tetrachloride. A Structural Isomer Containing Molybdenum-Molybdenum Interactions. Inorg. Chem. 7, 2091 (1968).

[b19] Larson M. & Moore F. Synthesis of Molybdenenum tetrachloride. Inorganic chemistry 3, 285 (1964).

[b20] Streltsov S. V. Orbital-selective behavior in Y5Mo2O12 and (Cd, Zn)V2O4. Journal of Magnetism and Magnetic Materials 383, 27 (2015).

[b21] Kimber S. A. J. *et al.* Valence bond liquid phase in the honeycomb lattice material Li$_2$RuO$_3$. Phys. Rev. B 89, 081408 (2014).

[b22] Sasioglu E., Friedrich C. & Blügel S. Effective Coulomb interaction in transition metals from constrained random-phase approximation. Physical Review B 83, 121101 (2011).

[b23] Anisimov V. I. *et al.* Calculation of the Coulomb Repulsion Parameter and Correlation Strength in Superconducting LaFeAsO. JETP Lett 88, 729 (2008).

[b24] Adams D. & Amadon B. Study of the volume and spin collapse in orthoferrite LuFeO$_3$ using LDA + U. Physical Review B 79, 115114 (2009).

[b25] Kuneš J., Korotin D., Korotin M., Anisimov V. & Werner P. Pressure-Driven Metal-Insulator Transition in Hematite from Dynamical Mean-Field Theory. Physical Review Letters 102, 146402 (2009). 19392460 10.1103/PhysRevLett.102.146402

[b26] McCarley R. & Brown T. The preparation and reactions of some tngsten(II) and tungsten(IV) halides. Inorg. Chem. 3, 1232–1236 (1964).

[b27] Sorantin P. I., Schwartz K., Sorantin P. I. & Schwartz K. Inorg. Chem. 31, 567 (1992).

[b28] Korotin M., Anisimov V., Khomskii D. & Sawatzky G. CrO$_2$: A Self-Doped Double Exchange Ferromagnet. Physical Review Letters 80, 4305 (1998).

[b29] Kim S., Kim K., Kang C.-J. & Min B. I. Pressure-induced phonon softenings and the structural and magnetic transitions in CrO_{2}. Physical Review B 85, 094106 (2012).

[b30] Giannozzi P. *et al.* QUANTUM ESPRESSO: a modular and open-source software project for quantum simulations of materials. Journal of Physics: Condensed Matter 21, 395502 (2009). 21832390 10.1088/0953-8984/21/39/395502

[b31] Perdew J. P., Burke K. & Ernzerhof M. Generalized Gradient Approximation Made Simple. Phys. Rev. Lett. 77, 3865 (1996). 10062328 10.1103/PhysRevLett.77.3865

[b32] Blöchl P. E. Projector augmented-wave method. Phys. Rev. B 50, 17953 (1994). 10.1103/physrevb.50.17953 9976227

